# Ultrasonic-assisted extraction, fatty acids identification of the seeds oil and isolation of chemical constituent from oil residue of *Belamcanda chinensis*

**DOI:** 10.1016/j.ultsonch.2022.106200

**Published:** 2022-10-14

**Authors:** Pu Liu, Shuang Kang, Yu-yang Huang, Tian-peng Song, Zi-yue Wu, Zong-yuan Lu, Rui-xue Deng

**Affiliations:** a Luoyang Key Laboratory of Natural Products Functional Factor Research and Development, Chemical Engineering & Pharmaceutical College, Henan University of Science and Technology, Luoyang, Henan 471023, China; b Queen Mary University of London Engineering School, Northwestern Polytechnical University, Xi’an, Shaanxi 710072, China; c Shanghai Standard Technology Co., Ltd., Pudong District, Shanghai 201314, China

**Keywords:** *Belamcanda chinensis* seeds, Ultrasonic-assisted extraction, Seed residue, Fatty acids, Iridal-type triterpenoids

## Abstract

*Belamcanda chinensis* is a common garden herb. The extraction technology of *B. chinensis* seed oil (BSO) was optimized by ultrasonic-assisted extraction (UAE) method, the composition, relative content of main fatty acids and physicochemical properties of BSO were determined, and the isolation, identification and determination of chemical constituent in BSO residue (BSOR) were also investigated. The optimum process conditions of BSO by UAE were optimized as ultrasound time 14 min, extraction temperature 42℃, the ultrasound power 413 W and the liquid–solid ratio 27:1 mL/g. Under this condition, the extraction yield was 22.32 % with the high contents of linoleic acid and oleic acid in BSO. Ten compounds were isolated and identified from BSOR, and belamcandaoid P (**9**) was a new compound. The contents of the determined compounds were all at high level in *B. chinensis* seed. The study provided a certain scientific reference for the comprehensive development and utilization of *B. chinensis* seeds.

## Highlights

• The chemical constituents of *B. chinensis* seed were systematically evaluated.
• The extraction conditions of BSO by UAE were optimized by RSM.
• UAE was a proper method for BSO extraction for the high yield (22.32%) and good quality.
• Much unsaturated fatty acids were identified in BSO.
• Three compounds were firstly isolated, belamcandaoid P was a new compound.

## Graphical abstract

**Figure:**
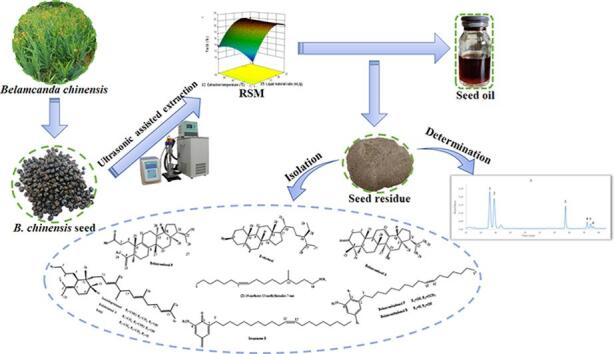

## 1 Introduction

*Belamcanda chinensis* (L.) Redouté, a perennial herb belonging to genus *Belamcanda* of Iridaceae, is a heliophilous plant and has the characteristic of cold resistance widely distributed in low-altitude forests, low-lying hillsides and grasslands of tropical, subtropical and temperate regions, especially in India, North Korea, Japan, Vietnam and China in Asia [1], [2], [3]. As a new type of common garden herb with both medicinal and ornamental value, *B. chinensis* is widely cultivated as an industrial crop in Northeast, North and West China [1], [4]. The rhizome of *B. chinensis* known was first recorded in *Divine Farmer*’*s Herb-Root Classic*, one of the oldest pharmacopoeias of traditional Chinese Medicine [5]. It had multiple effects such as clearing lung dust, benefiting the throat, evacuating congestion and reducing swelling and pain. As a traditional Chinese medicine, it mainly used to treat respiratory tract related inflammation, asthma, throat obstruction, carbuncle and sore poison, etc [1], [2], [3].

The seeds of *B. chinensis* contains oil and a variety of bioactive components, which has important edible value, could be used as source of edible functional oil [3], [4]. This paper carried out relevant systematic research on the chemical composition of *B. chinensis* seeds: the optimization of the ultrasonic extraction process of BSO, determination the composition and relative content of fatty acids (FAs) in BSO by GC–MS, evaluation the physical and physicochemical properties of BSO, systematically isolation and identification of the chemical components from BSOR, and determination the content of main active components in *B. chinensis* seeds by HPLC.

The main objective of the present work was to clarify the extraction conditions, the composition and relative content of main FAs of BSO, and the type and content of main active components in BSOR, so as to provide a certain scientific reference for promoting the comprehensive development and utilization of *B. chinensis* seeds, and also provide a new possible source for the development of natural food, medicine, beauty and daily chemicals from the *B. chinensis* seeds.

## 2 Materials and methods

### 2.1 Materials

The seeds of *B. chinensis* were obtained from Tuqiao Village Flowers and Trees Company, Mengjin District, Henan Province, China. The original plants and samples were identified by the pharmacist Hai-chen Liu of Luoyang Bencaotang Pharmaceutical Co., ltd. 50 g of voucher specimen each were deposited in School of Chemistry and Chemical Engineering, Henan University of Science and Technology (access number: 2022–015). The seeds of *B. chinensis* were crushed, sieved through 60 mesh sieve and kept at −4℃ for further use.

The ethyl acetate, acetone, petroleum ether (PE) (60-90℃) and *n*-hexane (analytical grade) were purchased from Tianjin Jindong Tianzheng Fine Chemical Reagent Factory (Tianjin, China).

### 2.2 The extraction of BSO

Ultrasonic-assisted extraction (UAE) was employed to extract the oil from *B. chinensis* seeds for the advantages of simple operation, short time and high efficiency [6]. A certain amount of dried seeds powder was weighed, and was put into a centrifuge tube, then added extraction solvent, the extraction was carried out at a set temperature and ultrasound power. After the extraction, the mixture was filtered through Whatman filter paper (0.45 μm) to get the extraction solution, then the oil was obtained by removing the solvent through rotary vacuum evaporator. The single factor experiments, along with the response surface methodology (RSM) were employed to optimize the extraction process conditions. The BSO yield could be obtained by the following equation.

$$\mathrm{Oil}\;yield\left(\%\right)=\left(\frac{\mathrm{weight}\;of\;extracted\;oil}{\mathrm{weight}\;of\;seeds}\right)\times 100\%$$

### 2.3 Analysis components of BSO by GC–MS

After esterification by the method described in GB/T 5009.168–2016 (National Standard of the People′s Republic of China) with NaOH-methanol, the composition and relative content of the FAs in BSO were determined by GC–MS according the conditions detailed described in literature [6]. The data collected by GC–MS were searched by NIST 2.2 standard spectrum library and compared with relevant literature to determine the main components of BSO, and the relative content was calculated by area normalization method.

### 2.4 Determination of physicochemical properties of BSO

The acid value (AV) (ISO2961, 1996), iodine value (IV) (ISO3961, 1996), peroxide value (POV) (ISO3960, 2001), saponification value (SV) (ISO3657, 2002) and unsaponifiable matter (USM) (ISO2596, 2002) were determined based on the International Organization for standardization (ISO).

### 2.5 The chemical constituent isolation and identification from BSOR

The dried powder of BSOR (10 Kg) was extracted with 40 L 90 % ethanol using reflux extraction method for 4 h for two times. The mixture was filtered to get the extraction solution, and extraction solution was concentrated by decompress concentration to obtain ethanol extract.

The ethanol extract of BSOR obtained above was suspended with water, and then extracted with PE, ethyl acetate (EtOAc) and *n*-butanol in the order of increasing polarity to obtain different fractions. Then, silica gel column chromatography (CC), LH-20 CC, semi-preparative HPLC and other chromatographic methods were employed to isolate the compounds from each fraction of PE, EtOAc and *n*-butanol.

The structures of the isolated compounds were identified by NMR, UV and MS.

### 2.6 Determination of main components in B. Chinensis seeds

According to the results of the chemical composition, the representative compounds, irisquinone E, belamcandaphenol P, belamcandaphenol B, iridal, isoiridogermanal and iridogelamal A were selected as the reference substance to determine the content of these main constituent in *B. chinensis* seeds by HPLC method.

#### 2.6.1 HPLC conditions

Waters e2695 HPLC system equipped with UV detector was used to determine the contents of the selected six compounds. The HPLC conditions were Thermo BDS HYPERSIL C18 (250 × 4.6 mm, 5 μm) column, column temperature 25℃, detection wavelength 254 nm, injection volume 10 μL, and the mobile phase of methanol (A)-0.1 % phosphoric acid (B) with gradient elution.

#### 2.6.2 Preparation of standard solution

The precisely weighed reference substance of irisquinone E (7.30 mg), belamcandaphenol P (9.60 mg), belamcandaphenol B (8.65 mg), iridal (6.5 mg), isoiridogermanal (5.53 mg) and iridobelamal A (5.9 mg) was dissolved in methanol to prepare the reference solutions with mass concentrations of 11.62 mg/mL, 2.40 mg/mL, 1.922 mg/mL, 1.625 mg/mL, 1.383 mg/mL and 1.475 mg/mL, respectively. The mixed reference stock solution containing irisquinone E (0.1296 mg/mL), belamcandaphenol P (0.1440 mg/mL), belamcandaphenol B (0.2306 mg/mL), iridal (0.1300 mg/mL), isoiridogermanal (0.1106 mg/mL) and iridobelamal A (0.1180 mg/mL) was prepared by added appropriate amount of the above reference solution into a 5 mL-volumetric flask and fixed the volume with methanol. The mixed reference stock solution with the volume of 0.167, 0.250, 0.334, 0.500, 1.000, 1.670 and 5.000 mL were added into 5 mL-volumetric flasks, respectively, and fixed the volume with chromatographic grade methanol to obtain standard solution with different concentration gradient.

#### 2.6.3 Sample solution preparation

The powder of BSOR (5 g) was extracted with methanol in a Soxhlet reflux device for 4 h. After extraction, the mixture was cooled, and then filtered. The filtrate was transferred into a 100 mL-volumetric flask and fixed the volume with methanol to get the sample solution. After filtration with an organic filter membrane (0.22 μm), the sample was determined.

## 3 Results and discussion

### 3.1 The selection of solvent for BSO extraction

Four samples of *B. chinensis* seeds powder with the same quality were extracted by Soxhlet extraction method with acetone, *n*-hexane, PE and EtOAc as solvents, respectively. The results revealed that the extraction yields of BSO with four solvents were as follows: acetone > *n*-hexane > PE > EtOAc. Compared with those using other solvents, the yield of BSO using acetone (26.01 %) was the highest. However, acetone is more toxic and thus less used in industry to extract oil. The yield of BSO extracted by EtOAc (20.63 %) was the lowest, and the color of the oil extracted by EtOAc was the deepest, which would increase the complexity of subsequent decolorization and other processes. The BSO yield extracted by PE (24.92 %) and *n*-hexane (25.42 %) was close, and with insignificant difference (*P* > 0.05). In addition, the color of BSO extracted by PE was better than that of the other three solvents, and the price of PE was lower than that of *n*-hexane. Therefore, PE was employed as the solvent in the follow-up oil extraction experiment considering the cost, toxicity and easy to obtain.

### 3.2 Optimization of extraction parameters of BSO

#### 3.2.1 The experiments of single factor

**Fig. 1:**
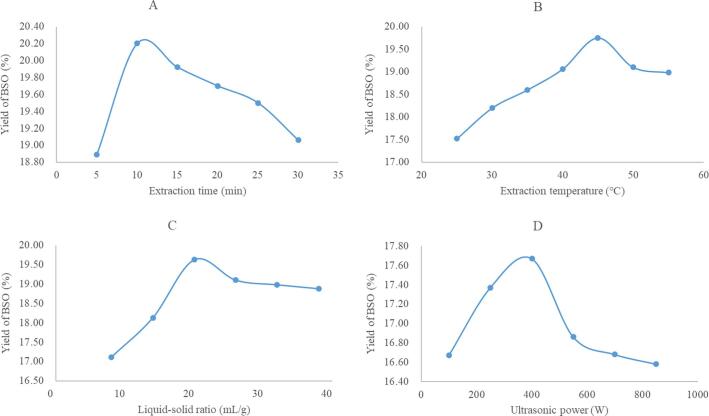
The effect of factor on yield of BSO.

Four factors such as extraction temperature, ultrasound power, ultrasound time and liquid–solid ratio were selected to optimize the effect of different factor on BSO yield. The results were shown in Fig. 1. It could be seen from the results that the influence trend of the four factors on the yield of BSO was basically the same, showing an increasing trend first and then decreasing. The yield of BSO reached the maximum when the extraction temperature, ultrasound power, ultrasound time and liquid–solid ratio at 45℃, 400 W, 10 min and 20:1 mL/g, respectively. Therefore, the factors and three levels of ultrasound time (5, 10, 15 min), liquid–solid ratio (14:1, 20:1, 26:1 mL/g), extraction temperature (40, 45, 50℃) and ultrasound power (250, 400, 450 W) were selected for the RSM experiments.

#### 3.2.2 The RSM design and statistical analysis

According to the single factor experiments results, four factors and three levels were selected for RSM experiments (Table 1). The Box-Behnken model was adopted to design the experiments, and the test was repeated at five central points. The results of 29 groups of RSM optimization experiments suggested that the yield of BSO varied from 11.36 % to 22.13 % (Table 1). The experimental data were analyzed by Design Expert 8.0.6 software (Table 1). Through multiple regression calculation, the fitting equation representing the relationship between the yield (*Y*) of BSO and ultrasound time (*X_1_*), ultrasonic extraction temperature (*X_2_*), liquid–solid ratio (*X_3_*) and ultrasound power (*X_4_*) was as follows:

$$\text{Y(\textbackslash{}\%)=21.14-0.43}X_{1}\text{-0.58}X_{2}\text{+3}{\text{X}}_{3}\text{+0.23}{\text{X}}_{4}\text{+0.058}{\text{X}}_{1}X_{2}\text{+2.75}{\text{X}}_{1}X_{3}\text{-0.62}X_{1}X_{4}+\text{0.35}X_{2}X_{3}\text{-0.29}X_{2}X_{4}\text{-0.43}{\text{X}}_{3}X_{4}\text{-0.34}{\text{X}}_{1}^{2}\text{-0.64}{\text{X}}_{2}^{2}\text{-3.62}{\text{X}}_{3}^{2}\text{-0.34}{\text{X}}_{4}^{2}\text{-}1.22X_{1}^{2}X_{3}+1.12X_{2}^{2}X_{3}$$

**Table 1:** Ultrasonic-assisted extraction RSM experiments design and results.

No.	*X_1_* (Ultrasound time, min)	*X_2_* (Extraction temperature, ℃)	*X_3_* (Liquid-solid ratio, mL/g)	*X_4_* (Ultrasound power, W)	*Yield* (%)	*Predicted*
1	−1(5)	−1(40)	0(20)	0(400)	21.88	21.22
2	0(10)	0(45)	1(26)	−1(250)	21.12	20.38
3	1(15)	−1(40)	1(26)	0(400)	21.79	21.22
4	1(15)	−1(40)	0(20)	0(400)	20.86	20.24
5	0(10)	0(45)	1(26)	1(550)	20.61	19.97
6	0(10)	0(45)	0(20)	0(400)	20.49	21.27
7	−1(5)	0(45)	0(20)	−1(250)	20.17	20.03
8	−1(5)	0(45)	−1(14)	0(400)	17.79	18.57
9	0(10)	0(45)	−1(14)	−1(250)	14.25	13.51
10	0(10)	1(50)	0(20)	−1(250)	19.09	19.63
11	0(10)	0(45)	0(20)	0(400)	20.81	21.14
12	0(10)	1(50)	0(20)	1(550)	19.22	19.51
13	0(10)	1(50)	1(26)	0(400)	20.43	20.76
14	0(10)	0(45)	0(20)	0(400)	21.13	21.14
15	1(15)	0(45)	−1(14)	0(400)	11.36	12.20
16	0(10)	−1(40)	0(20)	1(550)	20.18	21.26
17	0(10)	1(50)	−1(14)	0(400)	11.49	11.81
18	−1(5)	0(45)	1(26)	0(400)	15.84	16.62
19	0(10)	0(45)	0(20)	0(400)	22.13	21.14
20	1(15)	0(45)	0(20)	−1(250)	20.66	20.41
21	1(15)	0(45)	1(26)	0(400)	20.43	21.27
22	−1(5)	0(45)	0(20)	1(550)	21.74	21.74
23	0(10)	−1(40)	−1(14)	0(400)	14.25	13.51
24	0(10)	−1(40)	0(20)	−1(250)	18.88	20.21
25	1(15)	0(45)	0(20)	1(550)	19.75	19.64
26	0(10)	0(45)	−1(14)	1(550)	15.47	14.84
27	1(15)	1(50)	0(20)	0(400)	19.91	19.19
28	0(10)	0(45)	0(20)	0(400)	21.13	21.14
29	−1(5)	1(50)	0(20)	0(400)	20.70	19.94

The analysis of variance of RSM and the corresponding results were obtained (Table 2). The significance of four factors on the BSO yield by UAE could be determined according to the values of *P*-value and *F*-value. The *P*-value of < 0.01, between 0.01 and 0.05 and > 0.05 means extremely significant, significant and no-significant, respectively [6]. *F*-value can reflect the significance of the fitting degree of the experimental model equation. High *F*-value imply the good fitting degree of the model equation and high significance of the model [6]. The *P*-value (<0.0001) of liquid–solid ratio (*X_3_*) revealed that liquid–solid ratio had a significant effect on the yield of BSO. The influence order of each factor on the yield of BSO was liquid–solid ratio > extraction temperature > ultrasound time > ultrasound power (*X_3_* > *X_2_* > *X_1_* > *X_4_*). The *P*-values (≤0.0001) of interactive and quadratic terms *X_1_X_3_* and *X_3_^2^*, far<0.01, implied that the liquid–solid ratio and ultrasound time had a significant effect on BSO yield. At the same time, the *P*-value (<0.0001) of the RSM regression model and the *F*-value (14.89) revealed that the model was extremely significant. And the *P*-value (0.1174) of the lack of fit was far>0.05, indicating that the equation of the RSM regression model was good and could be employed to optimize the data in the experiment. The regression coefficient (*R^2^* = 0.9520) of this equation was close to the correction coefficient ($R_{\mathrm{Adj}}^{2}$ = 0.8881), which implied that the fitting degree of the model equation in this experiment was good. The value (12.814) of adeq precision of the model was>4, indicating that the precision of the experiment was good. All the results suggested that the predicted and experimental values of the model were within a reasonable range, and the model could be employed correctly to reflect the relationship between the yield of BSO and four factors [6].

**Table 2:** Response surface analysis of variance and results of UAE.

Source	Sum of squares	Degree of freedom	Mean square	*F*-value	*P*-value	Significance
Model	243.65	16	15.23	14.89	< 0.0001	**
*X_1_*	2.22	1	2.22	2.17	0.1662	
*X_2_*	4.06	1	4.06	3.97	0.0696	
*X_3_*	36.06	1	36.06	35.26	< 0.0001	**
*X_4_*	0.65	1	0.65	0.63	0.4419	
*X_1_X_2_*	0.013	1	0.013	0.013	0.9106	
*X_1_X_3_*	30.35	1	30.35	29.67	0.0001	**
*X_1_X_4_*	1.54	1	1.54	1.5	0.2437	
*X_2_X_3_*	0.49	1	0.49	0.48	0.5011	
*X_2_X_4_*	0.35	1	0.35	0.34	0.5717	
*X_3_X_4_*	0.75	1	0.75	0.73	0.4081	
*X_1_^2^*	0.77	1	0.77	0.75	0.4037	
*X_2_^2^*	2.69	1	2.69	2.63	0.181	
*X_3_^2^*	85.22	1	85.22	83.32	< 0.0001	**
*X_4_^2^*	0.74	1	0.74	0.72	0.4124	
*X_1_^2^X_3_*	2.99	1	2.99	2.92	0.1129	
*X_2_^2^X_3_*	2.5	1	2.5	2.44	0.1442	
Residual	12.27	12	1.02			
Lack of fit	10.76	8	1.35	3.56	0.1174	Not significant
Pure error	1.51	4	0.38			
Cor total	255.93	28				

*R^2^* = 0.9520, $R_{\mathrm{Adj}}^{2}$=0.8881, $R_{\mathrm{Pred}}^{2}$ = 0.5315, Adeq precision = 12.814, coefficient of variation (*C.V*) = 5.3 %, Sted. = 1.01, Mean = 19.09.

**Fig. 2:**
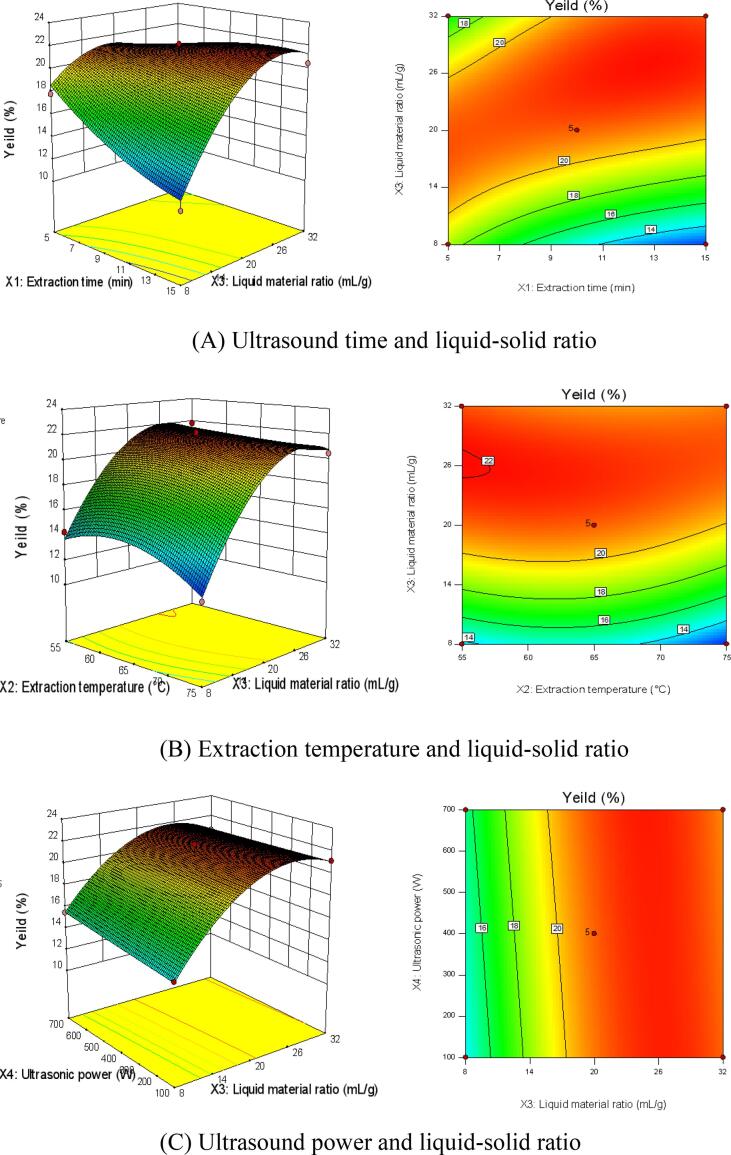
Response surface and contour lines for two-factor interaction.

According to the analysis of variance results of the model, the corresponding three-dimensional diagram and two-dimensional surface plots of RSM were obtained (Fig. 2). The influence of the interaction of the factors on the yield of BSO (*Y*) could be directly reflected from the diagram. The curvature of the three-dimensional surface plots could reveal the influence of the interaction of the factors on BSO yield. At the same time, the shape of the contour line corresponding to the three-dimensional surface plots could also reveal the strength of the interaction between the factors. When the contour line shape was close to elliptical, the interaction of factors was strong and the effect on the yield of BSO was more significant. By comparing the response surface plots and contour map of the interaction of various factors (A), (B) and (C) in Fig. 2, it could be seen that the effect of liquid–solid ratio (*X*_3_) and ultrasound time (*X*_1_) on BSO yield was the largest (Fig. 2 A), while the effects of liquid–solid ratio (*X*_3_) and ultrasound power (*X*_4_) on BSO yield was the smallest (Fig. 2 C). The results implied that the interaction between factors had the following effects on the yield of BSO: *X_1_X*_3_ > *X_2_X*_3_ > *X_3_X_4_*.

According to the analysis results of RSM model, the optimum conditions of UAE of BSO were as follows: ultrasound power 413 W, ultrasound time 14.12 min, liquid–solid ratio 27.34:1 mL/g, extraction temperature 41.61℃. Under the optimized conditions, the predicted BSO yield was 22.30 %. Considering the actual operation and instrument conditions, the optimum process conditions were adjusted to ultrasonic ultrasound time 14 min, ultrasonic extraction temperature 42℃, the ultrasound power 413 W and the liquid–solid ratio is 27:1 mL/g. Under this condition, the extraction yield of BSO was 22.32 %, and the relative deviation from the predicted value (22.30 %) was 0.08 %, indicating that the regression equation of the model could truly reflect the influence of various factors on the yield of BSO. The model was feasible for the optimization of UAE process of BSO.

The BSO was also extracted by Soxhlet extraction (SE) and impregnation extraction (IE) with PE as extraction solvent, and supercritical carbon dioxide (SC-CO_2_) according to the methods described in literatures [7], [8].The results revealed that the yields extracted by SE (25.68 %) and IE (23.96 %) were higher than that extracted by UAE (22.32 %), while the yields extracted by SC-CO_2_ (9.45 %) was lower than that extracted by UAE (22.32 %). The extraction time of SC-CO_2_ (180 min), SE (360 min) and IE (480 min) was much longer than that of UAE (14 min). SC-CO_2_ is a high-pressure extraction equipment, which requires high operating conditions and is difficult for industrial production [9]. So the extraction efficiency of SE, IE and SC-CO_2_ were much lower than that of UAE, and UAE was suitable for the extraction of BSO.

Ultrasonic-assisted extraction method has been widely used in the extraction of oil, showing a good extraction effect [10], [11], [12], [13], [14], [15]. The extraction rate of walnut oil by UAE (94.6 %) was higher than that by pressing method (60.68 %), SE (81.75 %), aqueous method (72.36 %) and SC-CO_2_ (91.24 %), and the extraction time (48 min) was much shorter than that by other extraction methods (130–360 min) [10]. The UAE yield of seed oil of *Prinsepia utilis* (32.5 %) was higher than that of SC-CO_2_ (31.9 %) and pressing method (28.2 %) [11]. The yield of oil from the fruits of *Nitraria sibirica* Pall by UAE (7.05 %) was higher than that by traditional extraction (5.24 %) and SE (5.73 %), and the extraction time (40 min) was much shorter than that of traditional extraction (360 min) and SE (480 min) [12]. It was found that the extraction time (50 min) by UAE of soybean germ oil was much shorter than that of SE (540 min) and SC-CO_2_ (180 min), and the oil yield by UAE (10.07 %) was higher than that by SC-CO_2_ (9.04 %) and slight lower than that by SE (10.56 %) [13]. The yields of peony seeds oil extracted by UAE (32.6 %) was higher than that by IE (31.5 %) and pressing method (23.5 %) [14]. The above results revealed that UAE could effectively shorten the extraction time, reduce energy and solvent consumption, and reduce carbon dioxide emissions. It has the advantages of energy saving, time saving and high efficiency [15].

### 3.3 Analysis of the composition and content of FAs of BSO

The components and relative contents of FAs in BSO extracted by Soxhlet method with different solvents, along with the BSO extracted by UAE, SE and IE with PE as extraction solvent, and SC-CO_2_ were determined by GC–MS (Figure S1). The results (Table 3) of the composition and relative content of FAs revealed that BSO was rich in a variety FAs, and linoleic acid was the prominent FAs with the highest content. No matter which solvent and which extraction method was used for the extraction of BSO, the relative content of linoleic acid (42.75–47.40 %) was higher than other FAs. The contents of oleic acid (33.63–37.81 %), palmitic acid (6.65–10.75 %) and stearic acid (3.08–5.35 %) were all at high levels. The total relative content of the above four FAs in BSO was 91.89–97.88 %, which implied that the four FAs were the major FAs in BSO. Among which stearic acid and palmitic acid were saturated fatty acids (SFAs), while linoleic acid and oleic acid were unsaturated fatty acids (UFAs). The total content of UFAs in BSO was 77.85–85.75 %, which was at a high level. Linoleic acid and oleic acid are common UFAs in vegetable oils, which play a very important role in maintaining the normal physiological activities of human and animals [16]. Oleic acid helps to soften blood vessels, regulate blood lipids, and is beneficial to the prevention of cardiovascular and cerebrovascular diseases [17]. Linoleic acid is an essential fatty acid component for human and animal nutrition. It could treat hyperlipidemia and arteriosclerosis, protect the liver, improve joint swelling and reduce rheumatoid arthritis [18], [19]. The content of linoleic acid (42.75–47.40 %) in BSO was higher than that of carrot seed oil (11.82 %) [20], chia seed (20.57 %) [21], and the common edible vegetable in China such as olive oil (3.5–21 %), palm oil (10.0–13.5 %), oil-tea camellia (3.8–14.0 %), flaxseed oil (10–20 %), peanut oil (13–43 %) and rice bran oil (19–42 %) (Table 4), and was lower than that of soybean oil (48–59 %), sunflower seed oil (48–74 %) and cottonseed oil (46.7–62.2 %), walnut oil (50–70), safflower seed oil (67.8–83.2 %) (Table 4), and watermelon seed (54.38 %), melon seed (56.39 %), pumpkin seed (63.75 %), sweet pumpkin seed (66.40 %), tomato seed (71.65 %) and red paprika seed (64.19 %) [22]. And the content of oleic acid (33.63–37.81 %) in BSO was higher than that of carrot seed oil (0.17 %) [20], chia seed (10.09 %) [21], and the common edible vegetable in China of the flaxseed oil (9.5–30 %), grapeseed oil (12–28 %), cottonseed oil (13.5–21.7 %), safflower seed oil (8.4–21.3 %), soya been oil (17–30 %) (Table 4), and watermelon seed (22.99 %), melon seed (22.94 %), pumpkin seed (15.36 %), sweet pumpkin seed (18.11 %), tomato seed (9.05 %) and red paprika seed (17.10 %) [22], and was lower than that of palm oil (39.8–46.0 %), oil-tea camellia (68–87 %), rice bran oil (40–50 %), peanut oil (36–69 %) and olive oil (55–83 %) (Table 4). It could be seen from the above research results that BSO was rich in UFAs and had an important edible value.

**Table 3:** Fatty acid composition and relative content (%) of BSO by different solvents and methods.

No.	Name	RT.	Solvent				Extraction method			
			P.E.	*n*-Hexane	Acetone	EtOAc	UAE	SE	SC-CO_2_	IE
1	Octanoic acid (C8:0)	6.21	0.05	0.18	0.13	0.14	0.02	0.05	0.04	0.16
2	Decanoic acid (C10:0)	9.31	0.03	–	0.05	0.06	0.03	0.03	0.01	0.07
3	Dodecanoic acid (C12:0)	11.96	0.02	–	0.03	0.03	–	0.02	0.05	0.23
4	Myristoleic acid (C14:1)	14.22	0.02	0.09	0.05	0.06	0.02	0.02	0.02	0.13
5	Myristic acid (C14:0)	14.37	0.55	1.32	1.57	1.67	0.28	0.55	0.49	1.83
6	Eicosanoic acid (C20:0)	15.11	0.16	–	–	0.11	–	0.16	0.21	2.13
7	*Cis*-6-octadecenoic acid (C8:0)	15.47	0.07	–	0.05	0.05	0.05	0.07	0.07	0.05
8	Pentadecanoic acid (C15:0)	15.92	0.05	–	0.04	0.04	0.04	0.05	0.05	0.05
9	Palmitoleic acid (C16:1)	17.54	0.55	0.41	0.92	1.11	0.39	0.55	0.59	1.16
10	Palmitic acid (C16:0)	18.03	8.76	8.97	10.53	10.75	6.65	8.76	9.94	10.55
11	14-methyl-hexadecanoic acid (C16:0)	19.87	0.07	–	0.05	0.07	0.04	0.07	0.05	0.06
12	2-hexyl-cyclopropaneoctanoic acid (C8:0)	19.99	0.12	–	0.07	0.08	0.08	0.12	0.13	0.07
13	*Cis*-10-heptadecenoic acid (C17:1)	20.23	0.03	–	0.05	0.13	0.02	0.03	0.03	0.06
14	Heptadecanoic acid (C17:0)	20.57	0.21	–	0.16	0.23	0.16	0.21	0.24	0.19
15	Linoleic acid (C18:2)	22.23	45.68	47.4	45.63	44.81	47.57	45.68	43.31	42.75
16	Oleic acid (C18:1)	22.37	37.81	37.09	34.65	33.63	37.53	37.81	37.81	33.41
17	Stearic acid (C18:0)	22.91	4.32	4.42	5.35	5.23	3.08	4.32	5.14	5.18
18	*Cis*-vaccenic acid (C18:1)	23.21	0.24	–	0.08	0.03	0.06	0.24	0.21	–
19	*Cis*-10-nonadecenoic acid (C19:1)	24.01	0.16	–	0.15	0.12	0.12	0.16	0.18	0.11
20	*Cis*-13-eicosenoic acid (C20:1)	25.32	0.09	–	0.21	0.24	0.04	0.09	0.08	0.23
21	17-methyl-octadecanoic acid (C18:0)	25.61	0.05	–	0.05	0.07	0.03	0.05	0.05	0.05
	Total content		99.22	99.88	99.88	98.66	96.21	99.22	98.84	98.47

**Table 4:** The physicochemical properties of edible vegetable oils are common in China.

Oils	IV	AV	POV	SV	USM	linoleic acid	oleic acid	Reference
Palm oil	50–55	≤10.0	≤5.0	190–209	≤12	10–13.5	39.8–46.0	GB 15680–2009
Sesame seed oil	104–120	≤4.0	≤7.5	186–195	≤20	36.9–47.9	34.4–45.5	GB 8233–2018
Maize oil	107–135	≤4.0	≤7.5	187–195	≤28	34–65.6	20–42.2	GB 19111–2017
Oil-tea camellia seed oil	83–89	≤4.0	≤7.5	193–196	≤15	3.8–14	68–87	GB 11765–2018
Flaxseed oil	165–208	≤3.0	≤7.5	188–195	≤15	10–20	9.5–30	GB 8235–2019
Grapeseed oil	118–141	≤3.0	≤7.5	188–194	≤15	58–78	12–28	GB 22478–2008
Cottonseed oil	100–115	≤1.0	≤6.0	189–198	≤15	46.7–62.2	13.5–21.7	GB 1537–2019
Rice bran oil	92–115	≤4.0	≤7.5	179–195	≤45	29–42	40–50	GB 19112–2003
Sunflower seed oil	118–141	≤4.0	≤7.5	188–194	≤15	48–74	14–39.4	GB 10464–2017
Peanut oil	86–107	≤4.0	≤7.5	187–196	≤10	13–43	36–69	GB 1534–2017
Walnut oil	140–174	≤3.0	≤6.0	183–197	≤20	50–70	11.5–35	GB 22327–2019
Soya been oil	124–139	≤4.0	≤6.0	189–195	≤15	48–59	17–30	GB 1535–2017
Safflower seed oil	136–148	≤4.0	≤7.5	186–198	≤15	67.8–83.2	8.4–21.3	GB 22465–2008
Olive oil	86–87	≤4.0	≤10	182–184	≤15	3.5–21.0	55–83	GB23347-2009

The composition and relative content results of the FAs of BSO extracted by four solvents suggested that the compositions of FAs in BSO extracted by *n*-hexane were less than those of the other three solvents, but the total FAs content (99.88 %) and the total content of the four major FAs (97.88 %) were slightly higher than those of other three solvents. The content of linoleic acid extracted by *n*-hexane (47.40 %) was the highest, while by EtOAc (44.81 %) was the lowest. The content of oleic acid extracted by PE (37.81 %) was the highest, while by EtOAc (33.63 %) was the lowest. The total content of linoleic acid and oleic acid extracted by PE (83.49 %) and *n*-hexane (84.49 %) were higher than those by acetone (80.28 %) and EtOAc (78.44 %). The above results implied that PE and *n*-hexane were the suitable solvents for extracting BSO. Considering the factors such as oil yield and solvent cost, PE is an ideal solvent for extracting BSO.

The content of linoleic acid extracted by UAE (47.57 %) was higher than that by SE (45.68 %), SC-CO_2_ (43.31 %) and IE (42.75 %), and the content of oleic acid by SE (37.81 %) and SC-CO_2_ (37.81 %) was higher than that by UAE (37.53 %) and IE (33.41 %). Which revealed that the total content of linoleic acid and oleic acid extracted by UAE (85.75 %) was higher than that by SE (83.49 %), SC-CO_2_ (81.12 %) and IE (76.16 %). And the content of UFAs in the seeds oil of *Prinsepia utilis* obtained by UAE was higher than that by SC-CO_2_ [11]. The content of linolenic acid and UFAs in peony seeds oil extracted by UAE (44.17 % and 70.42 %, respectively) was higher than that by IE (42.58 % and 69.62 %, respectively) and pressing method (43.89 % and 70.31 %, respectively) [14]. The above results suggested that the extraction methods had a relatively great influence on the relative content of major FAs in BSO. Due to the high content of UFAs in the extracted oil, UAE was a suitable method for extracting BSO.

### 3.4 Physicochemical properties of BSO

The AV (33.79 mg KOH/g), IV (105.44 g I_2_/100 g), POV (7.805 mmol/kg), SV (147.378 mg KOH/g) and USM (3.91 %) were determined by ISO methods. The physicochemical properties of common edible vegetable oils in China were shown in Table 4. The IV of BSO (105.44 g I_2_/100 g) was higher than that of olive oil (86–87 g I_2_/100 g), peanut oil (86–107 I_2_/100 g), oil-tea camellia seed oil (83–89 I_2_/100 g) and palm oil (50–55 I_2_/100 g), but was lower than that of maize oil (107–135 I_2_/100 g), flaxseed oil (165–208 I_2_/100 g), sesame seed oil (104–120 I_2_/100 g), grapeseed oil (118–141 I_2_/100 g), sunflower seed oil (118–141 I_2_/100 g), walnut oil (140–174 I_2_/100 g), soya been oil (124–139 I_2_/100 g) and safflower seed oil (136–148 I_2_/100 g). The results also implied that BSO had a high degree of unsaturation, the conclusion was consistent with the research results of high content of UFAs. The AV (33.79 mg KOH/g) was at high level, which was higher than that of most common vegetable oils (Table 4). High AV implied that the content of free FAs in BSO was high, BSO was prone to rancidity and had poor quality. The POV (7.805 mmol/Kg) was<10 mmol/Kg and was within the range specified in the National Standard of the People′s Republic of China. Compared with common edible oil in Table 4, the POV of BSO was still at a high level, which might be related to the oxidation of the double bond of UFAs of BSO in storage. The higher AV and POV indicated that the BSO must be refined before it could be used. SV can reflect the relative average molecular weight of oil, low SV indicates that the molecular weight of oil is large [23]. The SV of BSO (147.378 mg KOH/g) was lower than that of most common vegetable oils (Table 4), indicating that the molecular weight of BSO is at a high level. USM is an important index of vegetable oil quality, the high content of USM means the poor quality of the oil [24]. The USM (3.91 %) of BSO was within the permitted scope of common edible oil, which indicated that the quality of BSO was good.

The above physicochemical properties revealed that BSO should not be used as edible oil directly, and refining was a necessary step for the further processing and utilization of BSO. After further refining, BSO could be developed into edible oil, lubricating oil, daily chemicals, biodiesel and other industries.

### 3.5 The chemical constituent isolation and identification of BSOR

The extraction and separation process of BSOR after oil extraction was shown in Fig. 1. The dried BSOR (10 Kg) was extracted with 90 % ethanol, and extract (1200 g) was obtained. After suspension with water, the extract was extracted with PE, EtOAc and *n*-butanol, respectively, to obtain PE-fraction (520 g), EtOAc-fraction (15.5 g) and *n*-butanol-fraction (14.5 g).

**Fig. 3:**
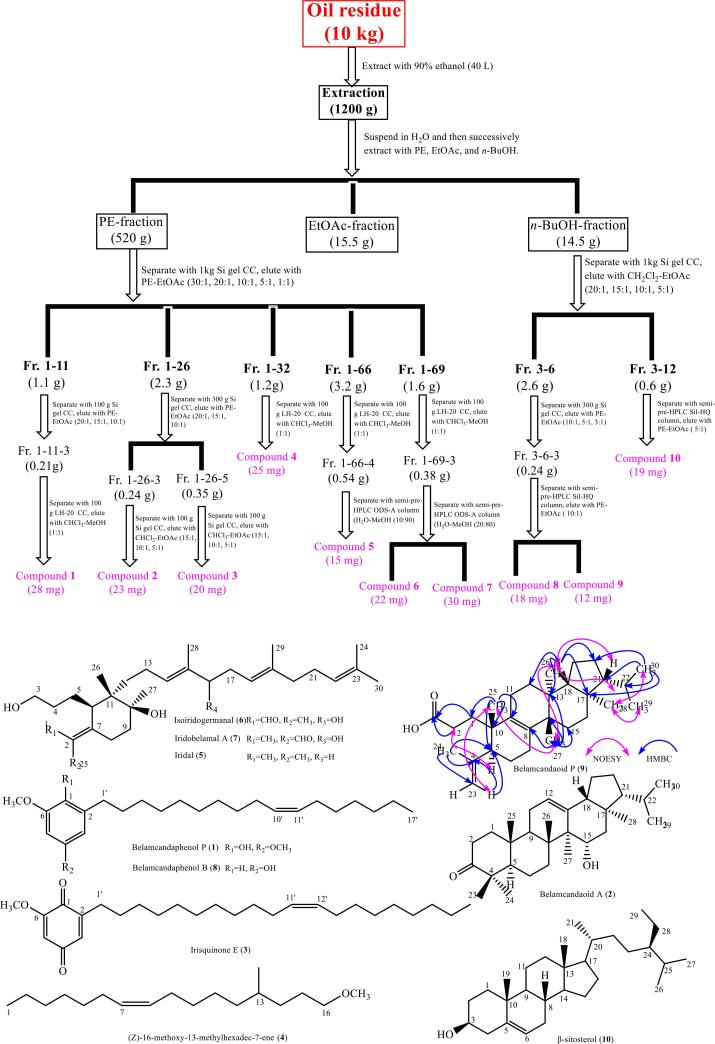
The extraction and isolation process, and the structures of the isolated compounds from BSOR.

The PE-fraction (100 g) was separated by silica gel CC, LH-20 CC and semi-preparative HPLC (semi-prep-HPLC) to obtain compounds **1**–**7**. Using the same methods, *n*-butanol-fraction (14.5 g) was separated to obtain compound **8**–**10**. The isolation process of the compounds was shown in Fig. 3.

The isolated compounds was identified as belamcandaphenol P (**1**) [25], belamcandaoid A (**2**) [4], irisquinone E (**3**) [26], (Z)-16-methoxy-13-methylhexadec-7-ene (**4**), iridal (**5**) [27], isoiridogermanal (**6**) [28], iridobelamal A (**7**) [29], belamcandaphenol B (**8**) [25], belamcandaoid P (**9**) and β-sitosterol (**10**) [30] by comparing the spectral data with that reported in the literatures, and two-dimensional NMR. Compound **9** was a novel one, compounds **5**–**7** were firstly isolated from *B. chinensis* seeds. The chemical structures of the ten compounds were shown in Fig. 3.

Compound **9** was obtained as a pale yellow acicular crystal. HR-ESI-MS [M + H]^+^ *m*/*z* 441.3732 (calcd: 441.3754 for C_30_H_49_O_2_), which revealed seven degrees of unsaturation of compound **9**. It could be seen from ^1^H NMR (CD_3_OD, 400 MHz) that compound **9** had two terminal olefinic proton signals (δ_H_: 4.7, 4.9) and 7 methyl protons, including 5 methyl singlet signals (δ_H_: 0.80 (3H, s), 0.83 (3H, s), 0.96 (3H, s), 0.99 (3H, s), 1.78 (3H, s)) and 2 methyl double peak signals (δ_H_: 0.85(3H, d, *J* = 4.8 Hz), 0.91(3H, d, *J* = 4.8 Hz)). The chemical shift of methyl (δ_H_: 1.78 (3H, s)), combined with the molecular formula of compound **9**, revealed that the methyl group (δ_H_: 1.78 (3H, s)) was connected to the double bond, which could be further confirmed through the HMBC cross-peaks of H-24 (δ_H_:1.78(3H, s)) with C-23(δ_C_: 113.5), C-4(δ_C_: 147.5) and C-5(δ_C_: 46.4), and H-23 (δ_H_: 4.7, 4.9) with C-4 (δ_C_: 147.5), C-5 (δ_C_: 46.4) and C-24 (δ_C_: 23.3). The H—H COSY cross-peaks of H-30 (δ_H_: 0.85(3H, d, *J* = 4.8 Hz)) with H-22 (δ_H_: 1.38(1H, m)), and of H-29 (δ_H_: 0.91(3H, d, *J* = 4.8 Hz)) with H-22 (δ_H_: 1.38(1H, m)), along with the HMBC cross-peaks of H-30 (δ_H_: 0.85(3H, d, *J* = 4.8 Hz)) with C-21 (δ_C_: 59.8), C-22 (δ_C_: 30.8) and C-29 (δ_C_: 23.0), and of H-29 (δ_H_: 0.91(3H, d, *J* = 4.8 Hz)) with C-21 (δ_C_: 59.8), C-22 (δ_C_: 30.8) and C-30 (δ_C_: 22.1) implied that there was an isopropyl structural fragment in the structure of compound **9**.

The ^13^C NMR (CD_3_OD, 100 MHz) of compound **9** revealed that there were 30 carbon signals, including a carboxyl carbon signal (δ_C_: 178.1), two double bond carbon signals (δ_C_:147.5, 113.5, 129.9, 138.5), and seven methyl carbon signals in high field (δ_C_: 23.33, 23.26, 23.0, 22.09, 21.75, 16.74 and 14.67). Through the analysis of carbon spectrum, hydrogen spectrum and two-dimensional NMR data of compound **9**, the spectral characteristics of compound **9** were consistent with those of the 3,4-*sec*-triterpenoids with fernane type, and were very similar to those of belamcandaoid B [4]. Therefore, it was preliminarily speculated that the compound **9** was of fernane type triterpenoids with 3,4-seco-structure.

The skeleton structure and main two-dimensional NMR correlation of compound **9** were shown in Fig. 3. Compared with belamcandaoid B, hydroxyl substitution signal and a methoxy signal was absence in compound **9**. In addition, the position of the double bond was also significantly different from that of belamcandaoid B. According to DEPT ^13^C NMR, the double bond carbon signals (δ_C_:129.9, 138.5) of compound **9** were quaternary carbon atom. The HMBC correlation from H-28 to C-8, H-25 to C-9, H-5 to C-8 and H-11 to C-8 confirmed that the double bond of compound **9** was at C8-C9 position. Unlike belamcandaoid B, the chemical shift of the carbonyl carbon signal in compound **9** obviously moved to the low field (δ_C_:178.1), indicating that the carbonyl group existed in compound **9** in the form of carboxylic acid rather than in the form of ester in belamcandaoid B. Which could be further confirmed by the high-resolution mass spectrometry of compound **9** and the reduced methoxy group in the signals. The spectral data of other positions of compound **9** could be further confirmed by two-dimensional spectrum (Fig. 3).

The configurations of spatial structure of compound **9** could be confirmed by NOESY spectrum. NOESY correlations such as H-25/H-26, H-27/H-18, H-21/H-18, H-27/H-28 all confirm that the spatial position relationship of compound **9** was consistent with that of belamcandaoid B: the configurations of the four positions of H-18(δ_H_ 1.58), H-25(δ_H_ 0.96), H-26(δ_H_ 0.83) and H-21(δ_H_ 1.01) were β-configuration, and H-27(δ_H_ 0.99) and H-28(δ_H_ 0.80) were α-configuration. The configuration at the corresponding positions (5S,10S,13R,14S,17R,18R,21R) were also confirmed. It was found that compound **9** had not been reported in the literature, so it was determined that this compound was a new triterpene, and named belamcandaoid P (**9**).

^1^H NMR(400 MHz, CDCl_3_) δ_H_: 4.9(1H, s, H-23a), 4.7(1H, s, H-23b), 2.44 (1H, m, H-2a), 2.20(1H, m, H-5), 2.18(1H, m, H-7a), 2.16(1H, m, H-2b), 2.12(1H, m, H-11b), 2.03(1H, m, H-16a), 1.96(1H, m, H-7b), 1.95(1H, m, H-16b), 1.83(2H, m, H-1), 1.78(3H, m, H-24), 1.70(1H, m, H-12a), 1.68(1H, m, H-15b), 1.64(2H, m, H-6), 1.58(1H, m, H-18), 1.54(1H, m, H-12b), 1.43(1H, m, H-19a), 1.38(1H, m, H-22), 1.29(2H, m, H-19), 1.01(2H, m, H-20), 0.99(3H, d, *J* = 4.0 Hz, H-27), 0.96(3H, d, *J* = 4.0 Hz, H-25), 0.91(1H, d, *J* = 1.2 Hz, H-30), 0.85(3H, s, H-29), 0.83(3H, t, *J* = 1.6 Hz, H-26), 0.80(3H, s, H-28); ^13^C NMR(100 MHz, CDCl_3_) δ_C_: 178.1 (C-3), 147.5(C-4), 138.5(C-8), 129.9(C-9), 113.5(C-23), 59.7(C-21), 52.7(C-18), 46.4 (C-5), 42.9(C-17), 41.8(C-15), 40.8(C-10), 36.5(C-13), 35.9(C-12), 32.1(C-1), 30.8(C-22), 30.5(C-16), 29.5(C-2), 29.3(C-20), 28.2(C-7), 26.9(C-16), 24.6(C-6), 23.3(C-25), 23.3 (C-24), 23.0(C-29), 22.1(C-30), 21.8(C-27), 21.6(C-11), 20.3(C-19), 16.7(C-26), 14.7 (C-28).

### 3.6 Determination of main chemical components in B. Chinensis seed

Compounds irisquinone E, belamcandaphenol B, belamcandaphenol P, iridal, isoiridogermanal and iridobelamal A with typical structural characteristics and high purity (>98 %) isolated from BSOR were selected as reference substance to determine the content of these compounds in *B. chinensis* seed.

The maximum absorption peaks of these compounds were recorded by wavelength scanning of UV, and the results revealed that the maximum absorption wavelength of all the compounds were at 230–270 nm. Based on the maximum absorption wavelength and the results in the literatures [31], [32], the wavelength (254 nm) was employed for the detection. Thermo BDS HYPERSIL C18 column (250 × 4.6 mm, 5 μm) was used at column temperature of 25℃. Methanol is a solvent commonly used in HPLC analysis. In this study, the solvent systems of methanol–water, methanol-formic acid water (0.1 %), methanol-phosphoric acid water (0.1 %) and methanol-phosphoric acid water (0.2 %) were evaluated for a good resolution and appropriate retention time. Finally, methanol (A)-0.1 % phosphoric acid (B) was selected as the mobile phase in gradient elution (Table S1). The effects of elution flow rate (0.3–1.0 mL/min) on the resolution, retention time and total analysis time of each component were successively investigated as well. The flow rate selected in this experiment changes with gradient, and the gradient change conditions were shown in Table S1.

The reference solution was analyzed with the above chromatographic conditions. The regression equation, precision, stability, repeatability, sample recovery, LOD (limit of detection)) and LOQ (limit of quantification) of the determination method were investigated according to the method literatures [33], [34]. The results were shown in Table 5. The research results revealed that the method had a very good linear relationship, and the precision, stability and repeatability of the method were very good. It could be used to determine the contents of these components in *B. chinensis* seed.

**Table 5:** Validation parameters of the developed method.

Compounds^a^	Calibration	Linear range µg.mL^−1^	*r*^2^	LOD µg/mL	LOQ µg/mL	Intra-day (%RSD)	Inter-day (%RSD)	Repeatability (%RSD)	Stability (%RSD)	Recovery (%)(%RSD)
**1**	y = 206106x-64.068	4.33–129.6	0.9995	0.80	2.40	1.54	1.20	0.86	0.38	99.90(0.11)
**2**	y = 54518x + 1947.3	4.81–144.0	0.9997	0.89	2.67	1.33	0.98	0.75	0.47	102.59(0.89)
**3**	y = 94637x + 1109.7	7.70–230.6	0.9992	1.42	4.27	2.01	1.66	0.28	1.53	98.58(0.82)
**4**	y = 652524x + 4270.6	4.34–130.0	0.9998	0.80	2.41	0.88	2.14	1.39	0.74	101.57(1.21)
**5**	y = 2104680x + 99800	3.69–110.6	0.9999	0.68	2.05	0.76	0.68	1.77	0.67	99.99(1.57)
**6**	y = 1616070x + 70406	3.94–118.0	0.9999	0.73	2.18	1.05	1.22	1.47	1.38	99.66(0.88)

a Numbers of standard compounds were isoiridogermanal (**1**), iridobelamal A (**2**), iridal (**3**), irisquinone E (**6**), belamcandaphenol B (**6**), belamcandaphenol P (**6**).

**Fig. 4:**
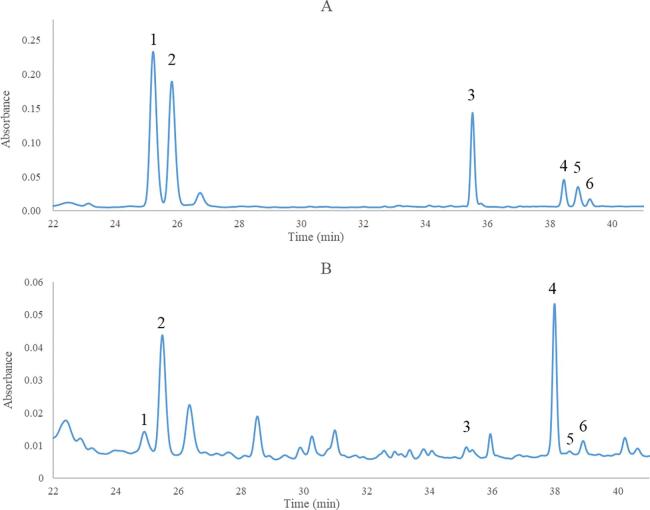
HPLC chromatograms (A- mixed reference substance, B-sample; 1-Isoiridogermanal, 2-Iridobelamal A, 3-iridal, 4-Irisquinone E, 5-Belamcandaphenol B, 6-Belamcandaphenol P).

The sample solution was determined according to the optimized HPLC chromatographic conditions (Fig. 4), Empower 3.0 software was used to treat the relevant data, and the contents of the components were calculated according to the corresponding peak area by external standard method. The results suggested that the contents of isoiridogermanal, iridobelamal A, iridal, irisquinone E, belamcandaphenol B and belamcandaphenol P in *B. chinensis* seed were 0.6139 ± 0.042, 1.0655 ± 0.032, 0.7058 ± 0.02, 9.8677 ± 0.048, 6.3042 ± 0.076 and 6.9585 ± 0.144 mg/g, respectively. Compound irisquinone E is benzoquinones, compounds belamcandaphenol B and belamcandaphenol P belong to phenols, and iridal, isoiridogermanal and iridobelamal A are iridal-type triterpenoids. Benzoquinones and phenols are common chemical components in *B. chinensis* seeds [3], which was further confirmed by the high content of irisquinone E, belamcandaphenol B and belamcandaphenol P in *B. chinensis* seed. Iridal-type triterpenoids, a kind of special compounds which come from the metabolism of squalene, are the representative components in the rhizoma of *B. chinensis* and Iridaceae plants with various and vital biological activities [2], [35]. This study revealed that the contents of isoiridogermanal, iridobelamal A and iridal were all at high level. A method for simultaneous determination of six main compounds in *B. chinensis* seeds was firstly established. At present, a variety of bioactive compounds had been isolated and identified from *B. chinensis*, flavonoids were mainly isolated in the roots and leaves of *B. chinensis*, and phenols, benzoquinones and volatile oil were mainly found in the seeds of *B. chinensis* [2], [3]. The chemical constituent isolation and identification on BSOR revealed that the contents of benzoquinones, phenols and iridal-type triterpenoids in *B. chinensis* seed were all at high level.

This study provided a foundation for the comprehensive development and utilization of *B. chinensis* seeds as a new resource food, and provided a scientific basis for the quality standard of *B. chinensis* seeds.

## 4 Conclusions

Overall, the extraction technology of BSO was optimized, the composition and relative content of main FAs and physicochemical properties of BSO were determined, and the isolation, identification and determination of chemical constituent in BSOR were also investigated in this study. The results revealed that UAE with PE as the extract solvent was the proper method for the extraction of BSO with the high yield (22.32 %) and good quality of the oil. The high content of oleic acid and linoleic acid makes BSO has an important potential application value as an edible oil. After further refining, BSO could be developed into edible oil, lubricating oil, daily chemicals, biodiesel and other industries.

Ten compounds were isolated and identified from BSOR, belamcandaoid P (**9**) was a new compound, and iridal, isoiridogermanal and iridobelamal A were firstly isolated from *B. chinensis* seed. The bioactive evaluation and the underlying pharmacological mechanisms may be further studied to promote the potential application of those compounds. The content of isoiridogermanal, iridobelamal A, iridal, irisquinone E, belamcandaphenol B and belamcandaphenol P were all at high level in *B. chinensis* seed. The results implied that in addition to rhizoma of *B. chinensis*, *B. chinensis* seed might also become a main source of iridal-type triterpenoids. The high oil yield, UFAs content, along with the abundant secondary metabolites make *B. chinensis* seed possible to become a new food resource. The further study is suggested to establish the standard of quality control, and evaluate the safety and security of potential products of *B. chinensis* seed. The study provided a certain scientific reference for promoting the comprehensive development and utilization of *B. chinensis* seed as a new resource food, and also provided a new possible source for the development of natural food, medicine, beauty and daily chemicals from the seed of *B. chinensis*.

## CRediT authorship contribution statement

**Pu Liu:** Methodology, Supervision, Visualization. **Shuang Kang:** Investigation, Writing – original draft. **Yu-yang Huang:** Investigation, Writing – original draft. **Tian-peng Song:** Investigation, Software. **Zi-yue Wu:** Investigation, Software. **Zong-yuan Lu:** Resources. **Rui-xue Deng:** Conceptualization, Supervision, Funding acquisition, Writing – original draft, Project administration.

## Declaration of Competing Interest

The authors declare that they have no known competing financial interests or personal relationships that could have appeared to influence the work reported in this paper.

## Data Availability

The data that has been used is confidential.
