# Supplementary material for: Ultrasonic-assisted extraction, fatty acids identification of the seeds oil and isolation of chemical constituent from oil residue of Belamcanda chinensis
Source: Ultrason Sonochem. 2022 Oct 14;90:106200. doi: 10.1016/j.ultsonch.2022.106200 (PMC9583576; doi:10.1016/j.ultsonch.2022.106200)
Supplement: Supplementary data 1 [file mmc1.docx]

**Table S1** HPLC mobile phase gradient condition.

| Time/min | Flow rate/mL.min^-1^ | Methanol (A%) | 0.1% phosphoric acid solution (B%) |
| --- | --- | --- | --- |
| 0 | 0.3 | 40 | 60 |
| 3 | 0.3 | 65 | 35 |
| 5 | 0.3 | 85 | 15 |
| 23 | 0.3 | 93 | 8 |
| 28 | 0.3 | 94 | 7 |
| 35 | 0.8 | 96 | 6 |
| 42 | 1.0 | 96 | 4 |
| 45 | 1.0 | 65 | 35 |
| 48 | 1.0 | 40 | 60 |

**Figure S1** The GC-MS chromatogram of BSO


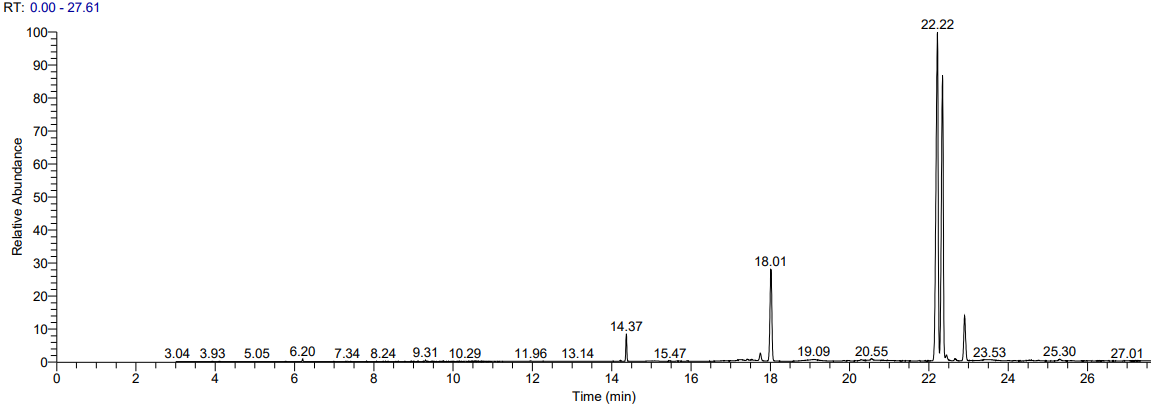


**The spectral data of the isolated compounds.**

Compound **1** (Belamcandaphenol P): Yellow oily liquid. ESI-MS m/z: 391 [M+H]^+^. ^1^H-NMR(400MHz, CDCl_3_) δ_H_: 6.38(1H, d, *J*=2.2 Hz, H-2), 6.32(1H, d, *J*=2.2 Hz, H-6), 5.36(1H, m, H-10'), 5.36(1H, m, H-11'), 5.32(1H, s, OH-4), 3.88(3H, s, OMe-3), 3.79(3H, s, OMe-1), 2.64(2H, t, *J*=8.0 Hz, H-1'), 2.04(4H, m, H-12', H-9'), 1.65(2H, q, *J*=8.0Hz, 4.0Hz, H-2'), 1.29(18H, m, H-3'~H-7', H-13'~H-16'), 0.90(3H, m, H-17'); ^13^C-NMR (400MHz, CDCl_3_) δ_C_: 152.71(C-1), 146.71(C-3), 137.48(C-4), 129.95(C-10'), 129.86(C-11'), 128.69(C-5), 105.67(C-6), 95.58(C-2), 55.96(OMe-3), 55.73 (OMe-1), 32.01(C-15'), 30.09(C-1'), 29.92(C-2'), 29.82(C-8'), 29.76(C-13'), 29.70(C-3'), 29.66 (C-4'), 29.62(C-5'), 29.62(C-6'), 29.36(C-7'), 29.36(C-14'), 27.2(C-9'), 26.96(C-12'), 22.4(C-16'), 14.06(C-17').

Compound **2** (Belamcandaoid A): Light yellow oily liquid. ^1^H-NMR(400MHz, CDCl_3_) δ_H_: 5.12(1H, m, H-12), 4.30(1H, t, *J*=4.0Hz, H-15), 2.57(1H, m, H-2a), 2.39(1H, dd, *J*=4.0Hz, 8.0Hz, H-16a), 2.37(1H, m, H-2b), 2.08(1H, m, H-11a), 2.06(2H, m, H-18), 1.90(1H, m, H-11b), 1.87(1H, m, H-20a), 1.86(1H, m, H-1a), 1.80(1H, m, H-9), 1.68(2H, m, H-7), 1.61(1H, m, H-19a), 1.56(2H, m, H-6), 1.47(1H, m, H-22), 1.46(1H, dd, *J*=4.0Hz, 8.0Hz, H-16b), 1.40(1H, m, H-1b), 1.33(1H, dd, *J*=12.0Hz, 4.0Hz, H-5), 1.25(2H, m, H-20b, H-21), 1.12(3H, s, H-27), 1.07(3H, s, H-24), 1.04(6H, s, H-23, H-25), 0.95(3H, d, *J*=8.0Hz, H-30), 0.88(3H, s, H-26), 0.86(3H, d, *J*=4.0Hz, H-29), 0.79(3H, s, H-28); ^13^C-NMR(400MHz, CDCl_3_) δ_C_: 17.54(C-3), 144.20(C-13), 119.24(C-12), 69.45(C-15), 60.07(C-21), 55.49(C-5), 51.89(C-18), 47.54(C-9), 46.83(C-16), 45.78(C-14), 41.02(C-8), 40.58(C-17), 38.75(C-1), 37.60(C-4), 37.62(C-10), 35.25(C-7), 34.11(C-2), 31.65(C-22), 28.26(C-20), 26.11(C-24), 23.61(C-11), 23.25(C-19), 22.53(C-29), 22.45(C-30), 21.37(C-23), 19.97(C-6), 17.93(C-28), 17.69(C-27), 16.88(C-26), 14.68(C-25).

Compound **3** (Irisquinone E): Reddish brown oily liquid. ^1^H-NMR(400MHz, CDCl_3_) δ_H_: 6.5(1H, dt, *J*=4.0Hz, 1.2Hz, H-3), 5.9(1H, d, *J*=4.0Hz, H-5), 5.37(1H, m, H-11'), 5.37(1H, m, H-10'), 3.84(3H, s, OMe-6), 2.45(2H, td, *J*=16.0Hz, 4.0Hz, H-1'), 2.01(4H, m, H-9', H-12'), 1.50(2H, m, H-2'), 1.28(14H, m, H-3'~H-8'), 1.28(12H, m, H-13'~H-18'), 0.91(3H, m, H-19'); ^13^C-NMR (400MHz, CDCl_3_) δ_C_：187.74(C-4), 182.18(C-1), 158.85(C-6), 147.59(C-2), 132.88(C-3), 129.90(C-10'), 129.87(C-11'), 107.11(C-3), 56.31(-OMe), 32.31(C-17'), 31.98(C-16'), 31.84(C-15'), 29.76(C-13'), 29.76(C-8'), 29.65(C-14'), 29.51(C-6'), 29.48(C-5'), 29.33(C-7'), 29.28(C-8'), 29.25(C-4'), 29.14(C-3'), 28.74(C-1'), 27.70(C-2'), 27.20(C-12'), 26.93(C-9'), 22.7(C-18'), 14.04(C-19').

Compound **4 (**(Z)-16-methoxy-13-methylhexadec-7-ene**)**: Purple amorphous powder. ^1^H-NMR(400MHz, CD_3_OD) δ_H_: 5.36(2H, t, *J*=8.0Hz, H-7/H-8), 3.84(3H, s, -OMe), 3.37(1H, s, H-16), 3.32(1H, m, H-16), 2.04(2H, m, H-6), 2.04(2H, m, H-5), 1.31(20H, m), 0.92(6H, m, H-1/H-17); ^13^C-NMR(400MHz, CD_3_OD) δ_C_: 129.45(C-7), 129.4(C-8), 55.82(OMe), 48.46(C-16), 31.75(C-3), 31.68(C-15), 29.45(C-4), 29.39(C-5), 29.31(C-10), 29.28(C-11), 29.17(C-13), 28.92(C-12), 27.88(C-14), 26.72(C-6), 26.49(C-9), 22.0(C-17), 21.96(C-2), 12.95(C-1).

Compound **5 (**iridal**)**: Light yellow oily liquid. ^1^H-NMR(400MHz, CD_3_OD) δ_H_: 10.2(1H, s, CHO), 5.11(1H, t, *J*=8.0Hz, H-18), 5.09(1H, t, *J*=8.0Hz, H-14), 5.07(1H, m, H-22), 4.99(1H, t, *J*=12.0Hz, H-17), 3.52(2H, t, *J*=8.0Hz, H-3), 3.37(1H, dd, *J*=12.0Hz, 1.2Hz, H-6), 2.6(1H, m, H-8a), 2.08(1H, m, H-8b), 2.06(2H, m, H-21), 1.98(2H, m, H-13), 1.96(1H, m, H-10), 1.83(2H, m, H-20), 1.83(3H, s, H-25), 1.62~1.69(2H, m, H-9), 1.68(3H, s, H-24), 1.62(3H, s, H-30), 1.58(3H, s, H-29), 1.53(3H, s, H-28), 1.331(2H, m, H-12), 1.31(2H, m, H-5), 1.14(3H, d, *J*=8.0Hz, H-27), 1.12 (3H, s, H-26); ^13^C-NMR(400MHz, CD_3_OD) δ_C_: 190.71(CHO-1), 165.3(C-7), 134.46(C-19), 134.43(C-2), 132.56(C-23), 130.66(C-15), 124.43(C-14), 124.06(C-18), 123.95(C-22), 73.9(C-17), 61.83(C-3), 44.7(C-16), 43.58(C-6), 39.49(C-11), 39.33(C-20), 37.06(C-10), 36.48(C-4), 32.33(C-9), 26.58(C-8), 26.43(C-21), 25.93(C-5), 24.55(C-24), 24.45(C-27), 23.63(C-13), 21.76(C-12), 17.01(C-30), 16.40(C-26), 14.76(C-29), 14.64(C-28), 9.44(C-25).

Compound **6 (**Isoiridogermanal**)**: Colorless oily liquid. ^1^H-NMR(400MHz, CD_3_OD) δ_H_: 10.21(1H, s, CHO-1), 5.25(1H, t, *J*=8.0Hz, H-12), 5.08(1H, m, H-16), 5.03(1H, t, H-20), 3.92(1H, t, *J*=8.0Hz, H-14), 3.52(2H, t, *J*=8.0Hz, H-25), 3.33(1H, m, H-5), 2.85(1H, dd, *J*=8.0Hz, 4.0Hz, H-9b), 2.66(1H, td, *J*=8.0Hz, 4.0Hz, H-9a), 2.23(3H, m, H-15), 2.06(2H, m, H-18), 2.04(1H, m, H-23b), 2.03(2H, m, H-18), 1.95(2H, m, H-11), 1.81(2H, m, H-8), 1.81(3H, m, H-3), 1.69(3H, s, H-22), 1.61(3H, s, H-29), 1.60(3H, s, H-30), 1.60(3H, s, H-28), 1.40(H, m, H-24b), 1.32(1H, m, H-10b), 1.14(H, m, H-24a), 1.12(1H, m, H-10a). ^13^C-NMR(400MHz, CD_3_OD) δ_C_: 191.23(1-CHO), 165.85(C-7), 136.25(C-17), 136.09(C-13), 132.53(C-2), 130.73(C-21), 126.34(C-12), 124.00(C-20), 120.22(C-16), 77.32(C-7), 74.00(C-14), 61.98(C-3), 47.67(C-6), 45.15(C-6), 39.50(C-18), 37.55(C-8), 36.77(C-10), 33.07(C-15), 31.77(C-4), 27.18(C-23), 26.43(C-5), 24.58(C-8), 24.49(C-22), 22.66(C-9), 19.78(C-11), 16.90(C-26), 16.44(C-30), 15.04(C-29), 10.93(C-28), 10.06(C-3).

Compound **7** (Iridobelamal A): Light yellow oily liquid. ^1^H-NMR(400MHz, CDCl_3_) δ_H_: 10.22(1H, s, CHO-1), 5.21(1H, t, *J*=8.0Hz, H-12), 5.09(1H, m, H-16), 5.02(1H, m, H-20), 3.87(1H, t, *J*=4.0Hz, 8.0Hz, H-14), 3.52(2H, t, *J*=8.0Hz, H-25), 3.32(1H, m, H-9a), 2.68(1H, td, *J*=12.0Hz, 4.0Hz, H-5), 2.59(1H, d, *J*=16.0Hz, H-9b), 2.13~2.23(2H, m, H-15), 2.10(1H, m, H-23b), 2.07(2H, m, H-19), 2.03(2H, m, H-18), 1.98(1H, m, H-11b), 1.90~1.96(1H, m, H-11a), 1.80(3H, m, H-3), 1.80(1H, m, H-23a), 1.79~1.72(2H, m, H-8), 1.69(3H, s, H-22), 1.62(3H, s, H-29), 1.59(3H, s, H-30), 1.59(3H, m, H-28), 1.33-1.36(2H, m, H-24), 1.18~1.31(2H, m, H-10), 1.14(3H, s, H-27), 1.13(3H, s, H-26). ^13^C-NMR(400MHz, CDCl_3_) δ_C_: 190.65(1-CHO), 165.15(C-4), 136.39(C-17), 136.04(C-13), 132.59(C-2), 130.74(C-21), 126.08(C-12), 124.01(C-20), 120.28(C-16), 77.3(C-14), 73.85(C-7), 61.83(C-25), 44.67(C-5), 43.61(C-6), 39.54(C-18), 36.82(C-8), 36.51(C-10), 33.09(C-15), 32.32(C-24), 26.59(C-23), 26.43(C-27), 24.58(C-19), 24.47(C-22), 23.63(C-11), 21.45(C-9), 17.00(C-26), 16.45(C-30), 15.04(C-29), 9.94(C-28), 9.50(C-3).

Compound **8** (Belamcandaphenol B): Reddish brown oily liquid.^1^H-NMR (400MHz, CDCl_3_) δ_H_: 6.35(1H, s, H-6), 6.28(1H, s, H-4), 6.26(1H, t, *J*=4.0Hz, H-2), 5.38(1H, m, H-10'), 5.37(1H, m, H-11'), 4.81(1H, s, OH-1), 3.79(3H, s, OMe-3), 2.53(2H, t, *J*=8.0Hz, H-1'), 2.04(2H, q, *J*=4.0Hz, H-9'), 2.04(2H, q, J=4.0Hz, H-12'), 1.65(2H, m, H-8'), 1.60(2H, q, *J*=8.0Hz, 12.0Hz, H-2'), 1.34(10H, m, H-3'~H-7'), 1.29(8H, m, H-13'~H-16'), 0.92(3H, m, H-17'). ^13^C-NMR(400MHz, CDCl_3_) δ_C_: 160.78 (C-3), 156.46(C-1), 145.81(C-5), 129.92(C-10'), 129.89(C-11'), 107.86(C-6), 106.77 (C-4), 98.62(C-2), 55.26(-OMe), 36.08(C-1'), 31.99(C-15'), 31.21(C-2'), 29.79(C-13'), 29.79(C-8'), 29.74(C-4'), 29.58(C-5'), 29.58(C-6'), 29.53(C-3'), 29.34(C-7'), 29.32(C-14'), 27.22(C-12'), 26.94(C-9'), 22.38(C-16'), 14.06(C-17').

Compound **10** (β-sitosterol)：White acicular crystal, ESI-MS m/z: 415 [M+H]^+^分子式为C_29_H_50_O. ^1^H-NMR(400MHz, CDCl_3_) δ_H_: 5.4(1H, d, *J*=2.66Hz, H-6), 3.6(1H, m, H-3), 1.95(2H, m, H-15), 1.8(3H, m, H-16), 1.45(10H, m), 1.1(3H, s, H-9/14/17), 1.0(3H, m, H-18), 0.9(3H, m, H-19), 0.85(3H, s, H-29), 0.8(3H, s, H-26), 0.7(3H, s, H-27). ^13^C-NMR (400MHz, CDCl_3_) δ_C_: 37.2(C-1), 32.89(C-2), 71.8(C-3), 42.4(C-4), 139.9(C-5), 121.8(C-6), 31.8(C-7), 50.5(C-8), 36.5(C-9), 40.2(C-10), 21.0(C-11), 32.0 (C-12), 42.3(C-13), 56.8(C-14), 24.2(C-15), 28.6(C-16), 56.1(C-17), 12.1(C-18), 19.9(C-19), 37.3(C-20), 19.2(C-21), 34.6(C-22), 26.4(C-23), 45.8(C-24), 29.7(C-25), 19.8(C-26), 19.2(C-27), 23.3(C-28), 12.2(C-29).
